# A case of esophageal neuromuscular choristoma

**DOI:** 10.1186/s12876-022-02249-2

**Published:** 2022-04-11

**Authors:** Wei Zhao, Xinying Zhu

**Affiliations:** 1 Department of Pathology, West China Second University Hospital, Sichuan University, Chengdu, China; 2 Key Laboratory of Birth Defects and Related Diseases of Women and Children (Sichuan University), Ministry of Education, Chengdu, China; 3 Department of Gastroenterology, The Third Hospital of Hebei Medical University, Shijiazhuang City, Hebei Province China

**Keywords:** Choristoma, Esophageal submucosal tumor, Benign triton tumor, Case report

## Abstract

**Background:**

Neuromuscular choristoma (NMC) is a rare peripheral nerve lesion that is composed of ectopic mature muscle fibers and nerve fascicles, typically involving major nerve roots or trunks, such as the cranial nerves, brachial plexus, and sciatic nerves. The onset of NMC frequently occurs in the first decade of life. Here, we present the first documented case of a case of esophageal NMC in an adult patient.

**Case presentation:**

A 46-year-old male patient presented in 2018 with a submucosal tumor of the esophagus. Upon presentation, the tumor was approximately 10 mm in diameter, covered by normal mucosa, and located in the left posterior wall of the esophagus in a position that was 30 cm from the incisor. The tumor was discovered incidentally during gastroscopic examination. In March 2021, endoscopic re-examination revealed no significant changes in the tumor. Endoscopic ultrasound revealed an oval hypoechoic mass with a homogeneous internal echo that originated from the muscularis propria with a maximum cross section of 13 mm × 6 mm. Resection was performed under gastroscopy. The resection specimen was 12 mm × 5 mm in size and was a well-demarcated, elastic, hard, and tough with a gray section. Histologically, the specimen consisted of an abundance of smooth muscle fiber bundles intercalated among nerve fibers, but without malignancy. Immunohistochemical examinations revealed positivity for S-100 protein, caldesmon, NSE and desmin, but negativity for CD117, DOG-1, HMB45, and Melan A. There was also aberrant nuclear localization of beta-catenin. Collectively, these findings led to a diagnosis of esophageal NMC.

**Conclusions:**

NMC is extremely rare, especially esophageal NMC, and is very challenging to accurately diagnose prior to resection. It is important that we can differentiate NMC from other types of tumors.

## Background

Neuromuscular choristoma (NMC), referred to as a ‘benign triton tumor’, is a rare benign nerve lesion that is characterized by differentiated muscles admixed with nerve fascicles [1–3]. The onset of NMC frequently occurs in the first decade of life [4–7]; only a small number of affected patients are adolescents or adults [8, 9]. The main symptoms of NMC are neurological abnormalities and changes in the soft tissue or bone [5].

Follow-up results relating to cases of NMC are limited and the exact characteristics and underlying mechanisms have yet to be clarified. It has previously been reported that NMC is frequently associated with desmoid-type fibromatosis (DTF) [10]. The etiological relationship between these two types of lesions may arise from CTNNB1- mutated (myo) fibroblasts within or immediately adjacent to the NMC [11]. Based on the association between these two diseases, patients with NMC should be constantly monitored, particularly with regards to the entire NMC-affected nerve. Cases of esophageal NMC have rarely been reported. Given the sporadic nature of esophageal NMC, it is particularly difficult to diagnose this disease in an accurate and timely manner. To the best of our knowledge, there is no mention in the existing literature of an adult case of esophageal NMC. Here, we report the first adult case of esophageal NMC, describe our experience with regards to diagnosis and treatment, and provide a brief review of NMC.

## Case presentation

**Fig. 1:**
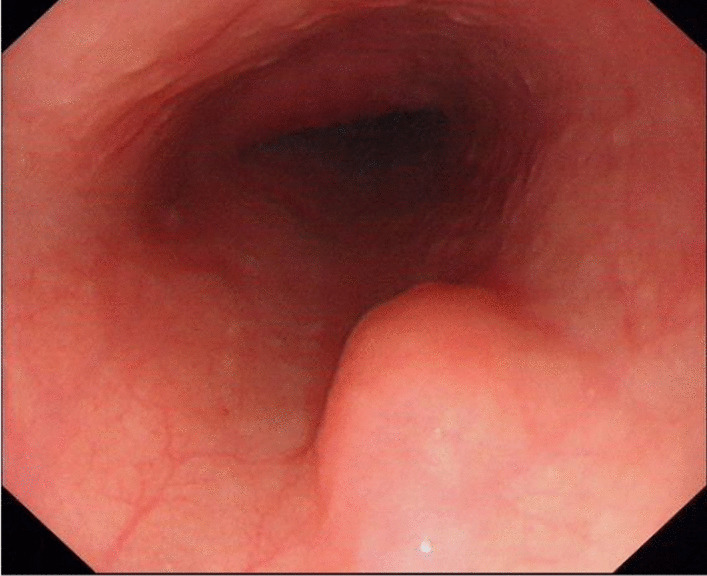
A submucosal tumor of the esophagus, about 10 mm in diameter, covered by normal mucosa, was discovered during gastroscopic examination

**Fig. 2:**
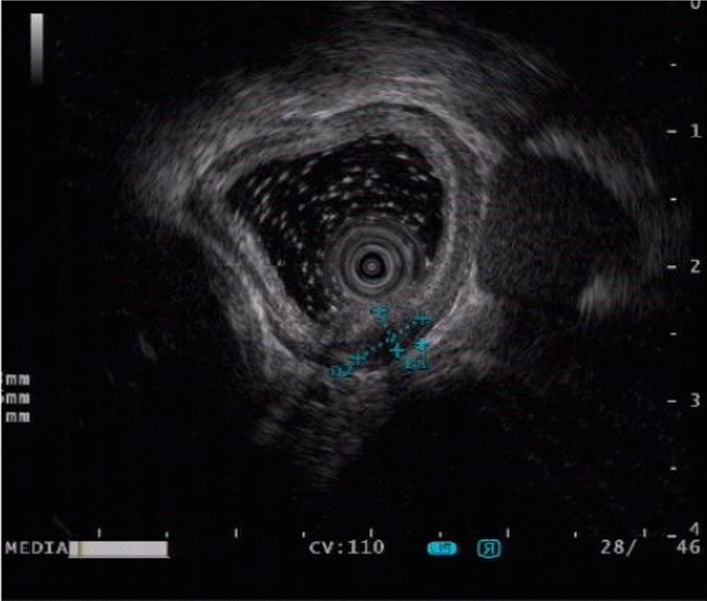
The ultrasound revealed an oval hypoechoic esophageal mass with homogeneous internal echo, originating from the muscularis propria, with a maximum cross section of 13 mm × 6 mm

A 46-year-old male presented with a submucosal tumor of the esophagus. In 2018, the tumor was approximately 10 mm in diameter, covered by normal mucosa, and located on the left posterior wall of the esophagus 30 cm from the incisor. The tumor was discovered by accident during gastroscopic examination (Fig. 1). The patient refused further evaluation and treatment due to the lack of clinical symptoms. In March 2021, endoscopic reexamination of the patient showed no significant changes in the tumor. There were no significant findings on physical examination. No special findings were found in terms of his medical, family, and psycho-social history. Laboratory tests of tumor markers in the patient’s serum were normal, including CEA and CA19-9. Endoscopic ultrasound revealed an oval hypoechoic mass with a homogeneous internal echo, originating from the muscularis propria, with a maximum cross section of 13 mm × 6 mm (Fig. 2). After learning that his neighbor had esophageal cancer, the patient felt compelled to undergo endoscopic resection to reveal the true pathological nature of his tumor as he was worried about the potential for malignancy.

**Fig. 3:**
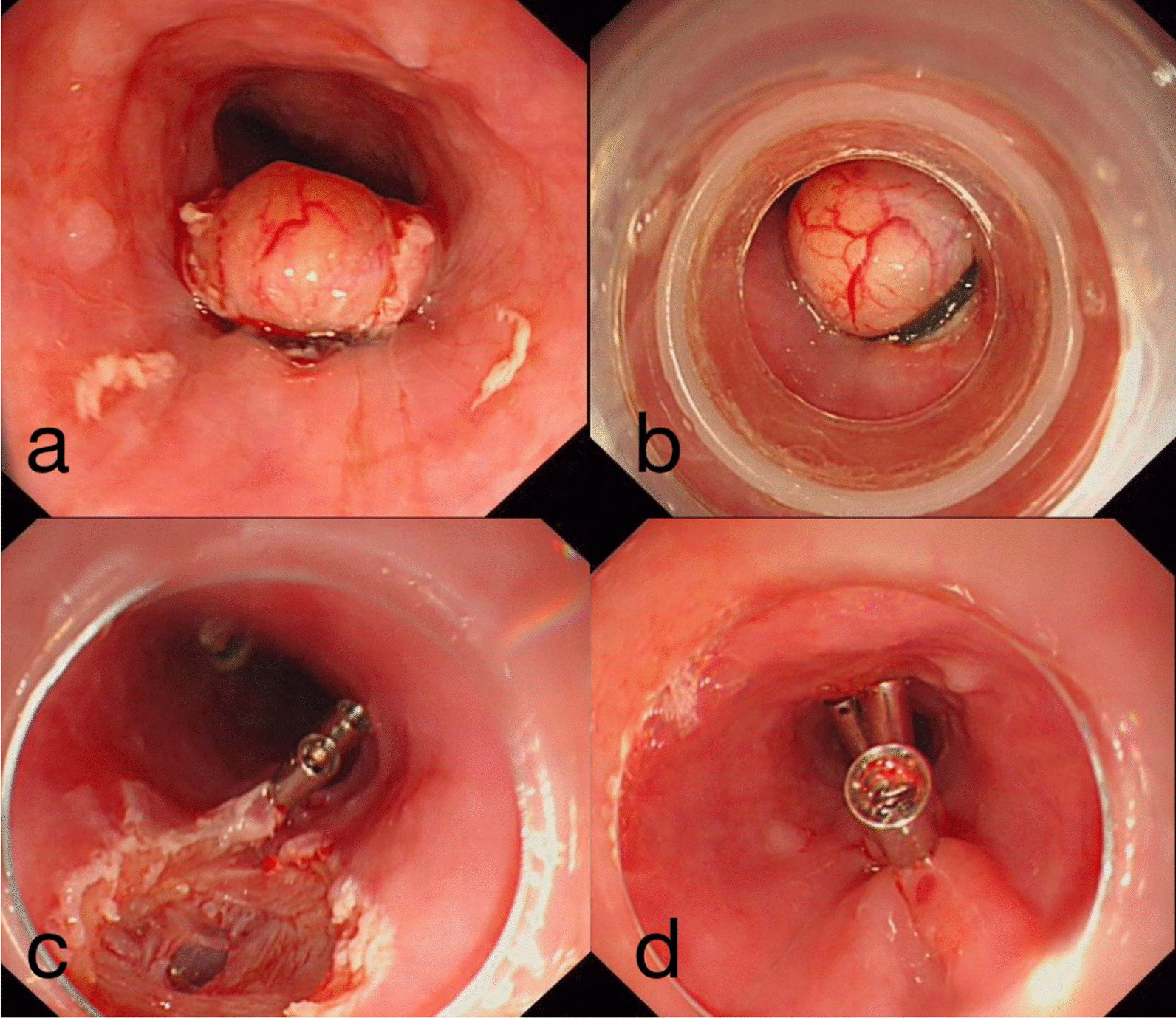
Endoscopic resection of the submucosal tumor. **a** The mucosa on the surface of the tumor was removed and the tumor was exposed. **b** The base of the tumor was ligated with a rubber band. **c** After the resection of the tumor, perforation of the esophageal wall was observed. **d** The perforation of the esophageal wall was sealed with metal clips

Gastroscopy revealed a smooth elevated lesion located on the left posterior wall of the esophagus 30 cm from the incisor teeth; the mucosa was intact. The mucosa on the surface of the tumor was removed by a snare and then the base of the tumor was ligated with a rubber band; subsequently, the tumor was excised by the snare. Following resection of the tumor, we observed perforation of the esophageal wall; this was sealed with metal clips (Fig. 3). Food and water were forbidden for 72 h after the operation, during which antibiotics, proton pump inhibitors, and intravenous nutrition were administered. The patient had no fever, hematemesis, melena, or dyspnea.

**Fig. 4:**
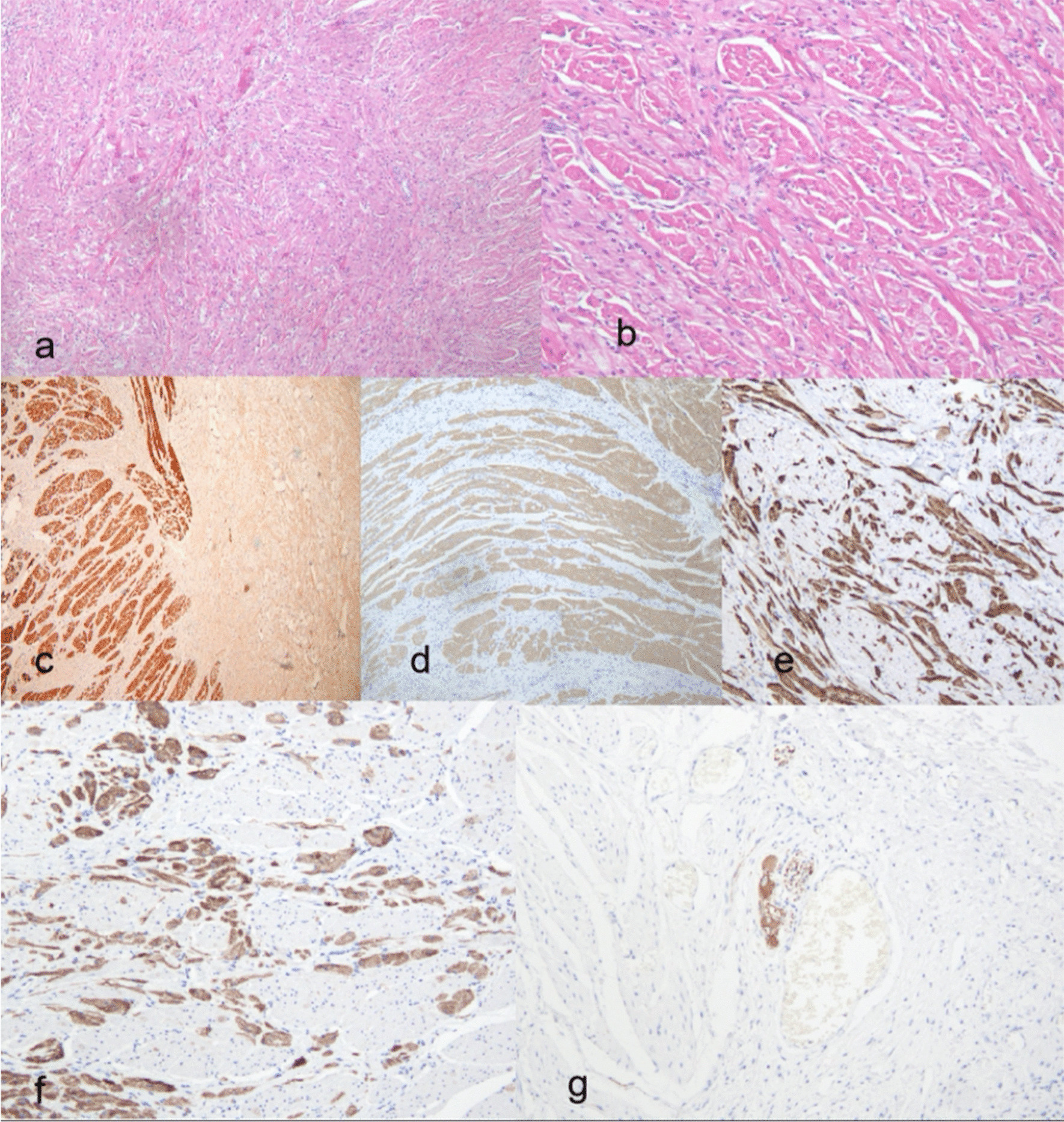
Photomicrographs of the resected tissue. Cross-section of the tumor is largely composed of bundles of muscle fibers intimately admixed with nerve fibers (**a**). Peripheral nerve bundles and strongly eosinophilic fibrous structures can be observed among myofascicles (**b**). Myofibers with various diameters in the interstitium were confirmed to be smooth muscle structures. strongly positive for desmin (**c**) and Caldesmon (**d**). S100 (**e**) and NSE (**f**) immunohistochemical stain highlights the embedded neurofibrils and small nerve fibers travelling through the muscle fibers. NF (**g**) immunohistochemistry stain is faintly labelled indicating the location of neurofilament

The surgical specimen was a well-demarcated, elastic, and hard mass that was 12 mm × 5 mm in size; the section of the specimen was gray and tough. Histologically, this lesion featured an abundance of smooth muscle fiber bundles intercalated among nerve fibers; there was no evidence of malignancy. Immunohistochemical examinations were positive for S-100 protein, caldesmon, neuron-specific enolase(NSE), Neurofilament(NF) and desmin. In addition, the lesion showed aberrant nuclear localization of beta-catenin and negativity for CD117, DOG-1, HMB45, and Melan A. Collectively, these data supported a diagnosis of esophageal neuromuscular choristoma (Fig. 4).

It has been 11 months since the resection of this esophageal submucosal tumor; the patient has no obvious clinical symptoms but has yet to be reviewed by gastroscopy.

## Discussion and conclusions

NMC is a rare peripheral nerve lesion that is composed of ectopic mature muscle fibers and nerve fascicles and typically involving major nerve roots or trunks [12, 13]. Since the first case of NMC was discovered in 1895 [14], approximately 53 cases of this lesion have been reported. This lesion is fairly common in the cranial, brachial plexus, or sciatic nerve [5, 15–17]. The major symptoms of patients with NMC are weakness, pain, deformity of the soft tissue or bone, and neurological abnormalities related the mass effect of variable duration, from months to years [7, 18, 19]. To the best of our knowledge, this is the first case of esophageal neuromuscular choristoma in an adult. Unlike intracranial neuromuscular choristoma and neuromuscular choristoma of the sciatic nerve, esophageal neuromuscular choristoma has no obvious clinical symptoms. Our patient had an esophageal space-occupying lesion that was detectable by physical examinations but without any clinical symptoms. Due to the lack of clinical symptoms, patients with esophageal NMC are not usually administered with appropriate treatment in a timely manner.

Patients with intracranial neuromuscular choristoma or neuromuscular choristoma of the sciatic nerve generally undergo imaging diagnostics [5, 18]. Magnetic Resonance Imaging (MRI) shows a well-circumscribed and fusiform enlargement of the affected nerve with characteristic T1 and T2-weighted signal nearisointense [20]. These lesions tend to appear with mild heterogenous enhancement on post-contrast images. It is not necessary to take MRI and Computed Tomography (CT) to diagnose esophageal illness, thus resulting in a lack of radiological findings for patients with esophageal NMC. No radiological examination was performed on our patient during either of his two appointments. Only ultrasound revealed an oval hypoechoic mass. In addition, blood tests failed to identify any other abnormal phenomena. Physicians find it very challenging to provide an accurate diagnosis at the initial visit. It remains highly challenging to diagnose NMC by standard imaging techniques; a definite diagnosis requires pathological examination with further immunostaining studies after resection. Under gastroscopy, we found a submucosal tumor of the esophagus that was 10 mm in diameter, covered by normal mucosa, and located on the left posterior wall of the esophagus 30 cm from the incisor teeth. When we found the lesion under gastroscopy, we were unable to establish a definitive diagnosis. We suspected an esophageal gastrointestinal stromal tumor, leiomyoma, or other benign form of mass lesion. For these reasons, we performed an extirpation of the lesion by application of a snare.

NMC has been considered as a hamartoma that featured the aberrant proliferation of peripheral nerve with a favorable outcome [4]. NMC associated desmoid-type fibromatosis (DTF) often arises in nerve territory affected by NMC [21, 22]. Moreover, histological examination of pathological specimens of aggressive fibromatosis previously revealed a close histological spatial association to NMC [23]. Some patients with DTF, but without a diagnosis of NMC, have been found to be occult cases of NMC [21]. It has been reported that the development of aggressive fibromatosis has a direct relationship with NMC and that patients with neuromuscular choristoma-associated DTF have been under reported. The etiology of NMC remains unclear. Mutation of the *CTNNB1* gene may be a common molecular genetic anomaly in NMC and NMC associated DTF. *CTNNB1* exon 3 mutations, which are identical to those observed in sporadic DTF, had been detected in 4 out of 5 NMC cases [11]. Based on existing data and clinical experience, patients with NMC need to be followed-up very closely (every 1–3 years). It is not yet known whether esophageal NMC is associated with the occurrence of aggressive fibromatosis. In view of the characteristics of NMC, physicians should consider NCM as a differential diagnosis when receiving and treating patients with esophageal occupying space so as to avoid missed diagnosis and improve imaging examinations such as CT and MRI.

Not all esophageal submucosal lesions require endoscopic removal/resection. In our current case, the esophageal submucosal tumor was discovered by accident at the age of 43; it is not known whether the lesion originated in infancy although the size of the tumor did not change significantly over the 3-year period, thus indicating an indolent characteristic. At initial diagnosis, a doctor told the patient that he could not have the lesion removed at that time, although regular endoscopic follow-up was required. Since there have been no previous reports of esophageal NMC, the natural course of this disease still needs to be investigated in a larger number of cases.

Here, we report the first case of NMC occurring in the esophagus of an adult. Although NMC is widely perceived as a benign disease, it should be included in the differential diagnosis of neoplastic lesions not only in the cranial, brachial plexus, or sciatic nerves, but also in the esophagus. Patients with esophageal NMC also need to be followed-up closely.

## Data Availability

The patient’s data and images can be found in the database of Information Office of Third Hospital of Hebei Medical University.

## Abbreviations

NMC: Neuromuscular choristoma
DTF: Desmoid-type fibromatosis
CEA: Carcinoembryonic antigen
CA: Carbohydrate antigen
CD: Cluster of differentiation
